# Focal Arterialization and Neoatherosclerosis of a Saphenous Vein Graft. Improving our Understanding of Late Graft Failures

**DOI:** 10.5935/abc.20160172

**Published:** 2016-11

**Authors:** D Rene Hameau, P Nicolas Veas, L Manuel Mendez, R Gonzalo Martinez

**Affiliations:** Hospital Clínico Pontificia Universidad Católica de Chile, Santiago - Chile

**Keywords:** Myocardial Revascularization, Saphenous Vein, Atherosclerosis, Tomography, Optical Coherence

**Figure 1:**
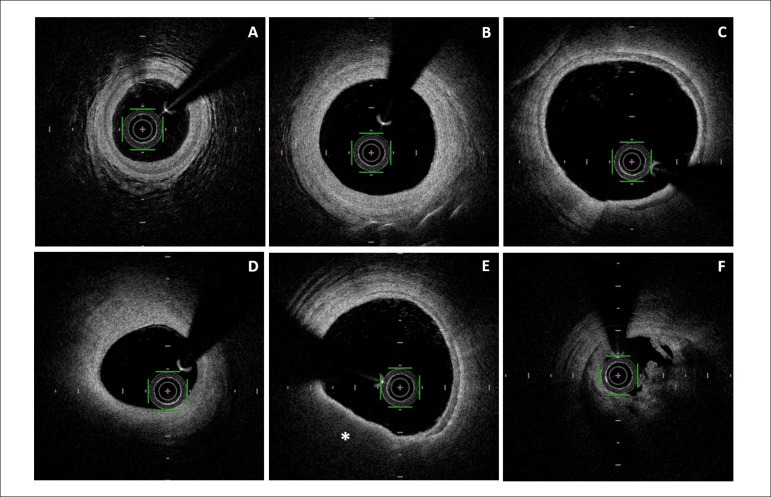
Optical coherence tomography of the saphenous vein graft (SVG) to first diagonal. A) Native diagonal coronary artery, showing distinct three-layer wall; B) Mildly diseased distal segment of SVG; C) Arterialized segment of the SVG, showing remarkable similarity to native coronary artery anatomy; D) Significantly diseased segment of SVG; E) Arterialized segment of SVG, harboring neoatherosclerosis in form of a thin cap fibroatheroma (*); F) Thrombotic critical lesion in the proximal segment of the SVG.

A 76 year-old man with a past medical history of type 2 Diabetes, stage 3 chronic kidney disease and previous multivessel coronary artery bypass surgery 10 years before was admitted with an acute coronary syndrome. Coronary angiogram showed an occluded saphenous vein graft (SVG) to first diagonal. After restoring flow by means of thrombus aspiration, optical coherence tomography (OCT) runs were required. Localized and widely patent segments of the graft showed a clear three-layer configuration, suggestive of SVG focal arterialization. Proximally, predominantly red thrombus determined a critical stenosis and a thin cap fibroatheroma was evident in an arterialized segment of the graft contiguous to the thrombotic lesion. Conversely, the rest of the SVG kept its venous appearance and had three different areas of stenosis (Figure 1).

Since the early 70´s, there has been multiple efforts to describe and understand factors determining SVG patency. Remodeling - mainly through intimal hyperplasia and further neo atherosclerosis - is responsible for most of the late graft loss.

The present case adds to what is previously known by showing distinct segments of three-layered vein graft wall, and also that SVG "arterialization" can be a focal and heterogeneous process throughout the vessel wall. Furthermore, it was a vulnerable plaque from this remodeled segment the one that probably harbored the thrombotic complication.

Given its superior image resolution, OCT can significantly contribute to our understanding of vein graft failures.

